# Draft Genome Sequences of *Staphylococcus aureus* Strains Isolated from Subclinical Bovine Mastitis in Brazil

**DOI:** 10.1128/genomeA.01594-15

**Published:** 2016-02-18

**Authors:** Danielle Mendes Silva, Mônica Pacheco da Silva, Pedro M. Pereira Vidigal, Rafael Mazioli Barcelos, Raphael Contelli Klein, Ananda Pereira Aguilar, Mary Hellen Fabres-Klein, Guilherme Oliveira, Andréa Oliveira Barros Ribon

**Affiliations:** a Departamento de Bioquímica e Biologia Molecular, Centro de Ciências Biológicas, Universidade Federal de Viçosa, Minas Gerais, Brazil; b Núcleo de Análise de Biomoléculas (NuBioMol), Centro de Ciências Biológicas, Universidade Federal de Viçosa, Minas Gerais, Brazil; c Centro de Pesquisas René Rachou (CPqRR)—FIOCRUZ, Belo Horizonte, Minas Gerais, Brazil; d Vale Technology Institute, Belém, Paraná, Brazil

## Abstract

Here, we present the draft genome sequences of four *Staphylococcus aureus* strains isolated from mastitic milk collected from animals with subclinical manifestations. Three of them were typed as sequence type 126 (ST126), a genotype with no genome sequence available. ST126 is found in several herds of southern Brazil and is described as a bovine pathogen strongly associated with milk around the world.

## GENOME ANNOUNCEMENT

*Staphylococcus aureus* is one of the main pathogens isolated from bovine mastitis infections (1). Intense efforts have been made to understand the molecular mechanisms of bacterial pathogenesis (2, 3) and to link bacterial characteristics with the specific clinical manifestations of bovine mastitis (4). These strain-specific markers could be used to track relevant strains in herds that would positively affect animal health and welfare.

We monitored two herds in the State of Minas Gerais, Brazil, for the presence of subclinical mastitis for 6 months. Bacteria identified as *S. aureus* were genotyped by multilocus variable-number tandem-repeat analysis (MLVA) and pulsed-field gel electrophoresis (PFGE). Four isolates were selected for genome sequencing. SAU-302 and SAU-1364 were isolated from two cows with a persistent subclinical infection, while SAU-170 and SAU-1269 were isolated from two cows with subclinical infections for a month. The infection was considered persistent if it was detected after three or more consecutive months from the same animal.

Four 200-bp single-end genomic libraries were constructed and sequenced on an Ion Torrent Personal Genome Machine (PGM). Four data sets were generated, containing 1,422,030 reads (SAU-170), 1,250,612 reads (SAU-302), 2,572,399 reads (SAU-1269), and 634,729 reads (SAU-1364). The sequenced reads were trimmed for length (minimum, 100 bp) and quality (minimum score, Q20) and *de novo* assembled to contigs using CLC Genomics Workbench version 6.5.1 (CLC bio). This assembly produced 194 contigs and overall G+C content of 32.8% for SAU-170, 568 contigs and 32.9% G+C content for SAU-302, 93 contigs and 32.7% G+C content for SAU-1269, and 287 contigs and 32.7% G+C content for SAU-1364. Genes were predicted from the contigs using Prodigal version 2.50 (5), which revealed the coding sequence (CDS) set of SAU-170 (2,599 CDSs), SAU-302 (2,666 CDSs), SAU-1269 (2,545 CDSs), and SAU-1364 (2,547 CDSs). The protein sets were functionally annotated using BLAST searches (http://blast.ncbi.nlm.nih.gov/), and approximately 77% of the proteins of each strain were assigned Clusters of Orthologous Groups (COG) families (6).

In genotyping analysis, SAU-170, SAU-302, and SAU-1269 were classified as sequence type 126 (ST126), and SAU-1364 was classified as ST1 by multilocus sequence type (MLST) analysis (http://saureus.mlst.net/misc/info.asp). ST1 is a type isolated from bovine and human infections (7). ST126 is a prevalent genotype found in several herds in southern Brazil (8, 9) and was described elsewhere as a bovine pathogen strongly associated with milk (7). We also genotyped the strains against the reference genome of *S. aureus* RF122 (accession no. NC_007622), a strain representative of the major clone involved in severe bovine mastitis worldwide (10). We identified single-nucleotide polymorphisms (SNPs) that resulted in amino acid changes in the coded protein. A total of 6,273 SNPs were detected in SAU-170, 5,481 in SAU-302, 7,420 in SAU-1269, and 2,491 in SAU-1364 (CLC bio; minimum, 20X coverage [11]). These new genomes add information to the repertoire of genes described for strains associated with subclinical mastitis that will be useful in studies to elucidate molecular mechanisms of pathogenesis.

### Nucleotide sequence accession numbers.

The draft genome sequences of SAU-170, SAU-302, SAU-1269, and SAU-1364 are available in GenBank under the accession numbers LNOQ00000000, LNOR00000000, LNOO000000000, and LNOP00000000, respectively.
