# Erratum for Silva et al., Draft Genome Sequences of *Staphylococcus aureus* Strains Isolated from Subclinical Bovine Mastitis in Brazil

**DOI:** 10.1128/genomeA.00350-16

**Published:** 2016-04-21

**Authors:** Danielle Mendes Silva, Mônica Pacheco da Silva, Pedro M. Pereira Vidigal, Rafael Mazioli Barcelos, Raphael Contelli Klein, Ananda Pereira Aguilar, Mary Hellen Fabres-Klein, Guilherme Oliveira, Andréa Oliveira Barros Ribon

**Affiliations:** a Departamento de Bioquímica e Biologia Molecular, Centro de Ciências Biológicas e da Saúde, Universidade Federal de Viçosa, Minas Gerais, Brazil; b Núcleo de Análise de Biomoléculas (NuBioMol), Centro de Ciências Biológicas e da Saúde, Universidade Federal de Viçosa, Minas Gerais, Brazil; c Centro de Pesquisas René Rachou (CPqRR)—FIOCRUZ, Belo Horizonte, Minas Gerais, Brazil; d Vale Institute of Technology, Belém, Pará, Brazil

## ERRATUM

Volume 4, no. 1, e01594-15, 2016. Page 1: The affiliation line should read as given above.

